# Supplementary material for: Size-Dependent Effects of Polystyrene Nanoparticles (PS-NPs) on Behaviors and Endogenous Neurochemicals in Zebrafish Larvae
Source: Int J Mol Sci. 2022 Sep 14;23(18):10682. doi: 10.3390/ijms231810682 (PMC9505408; doi:10.3390/ijms231810682)
Supplement: Supplementary file 1 [file ijms-23-10682-s001.zip › ijms-1898653-supplementary.pdf]

Supplementary Information

Size-Dependent Effects of Polystyrene Nanoparticles (PS-NPs) on Behaviors and Endogenous Neurochemicals in Zebrafish Larvae

Kyu-Seok Hwang †, Yuji Son †, Seong Soon Kim, Dae-Seop Shin, So Hee Lim, Jung Yoon Yang, Ha Neul Jeong, Byung Hoi Lee and Myung Ae Bae *

Therapeutics & Biotechnology Division, Korea Research Institute of Chemical Technology, Daejeon 34114, Korea

* Correspondence: mbae@krict.re.kr.

† These authors contributed equally to this work.

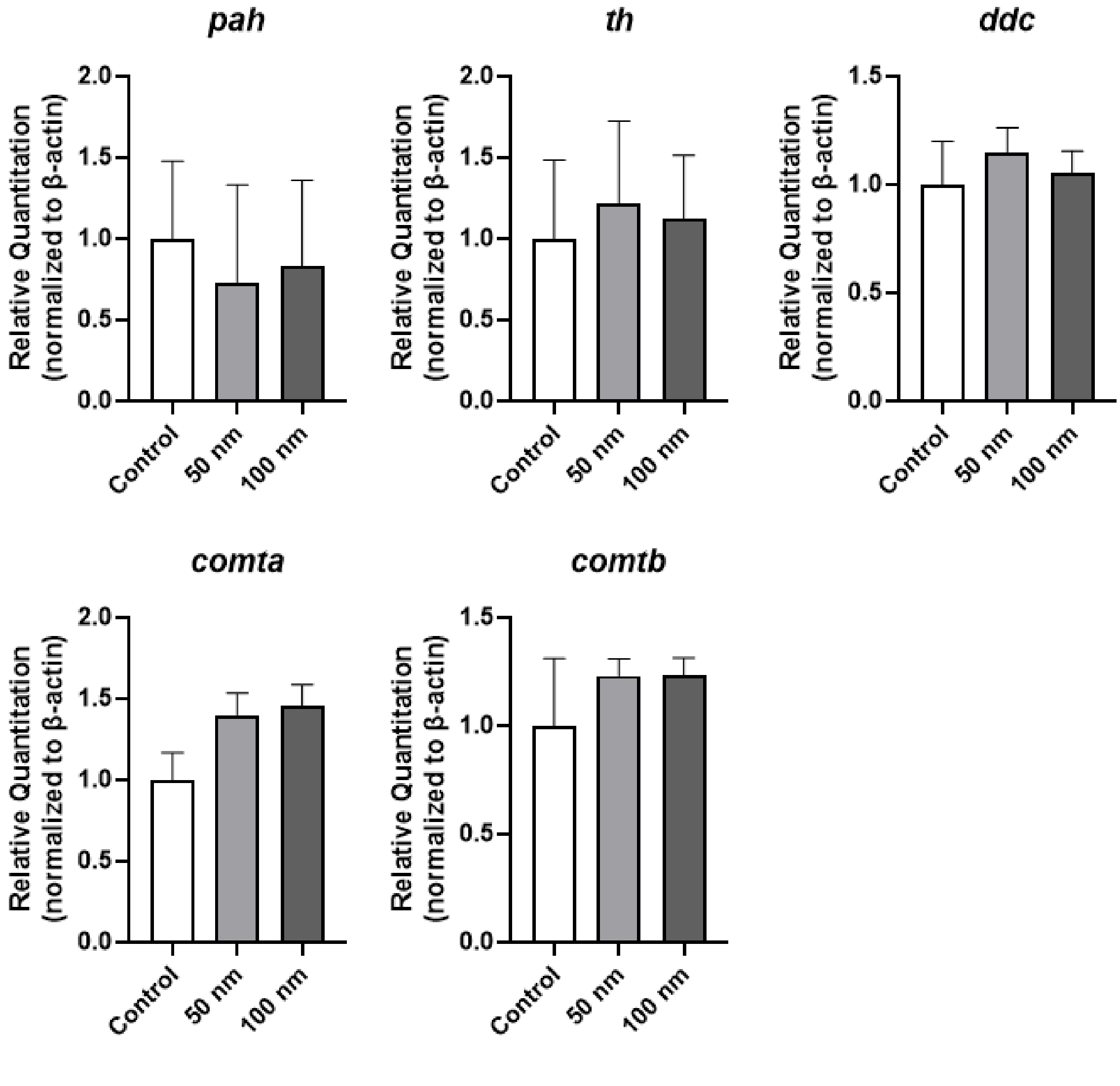

Figure S1. Analysis of gene expression for dopaminergic metabolite-related enzymes.

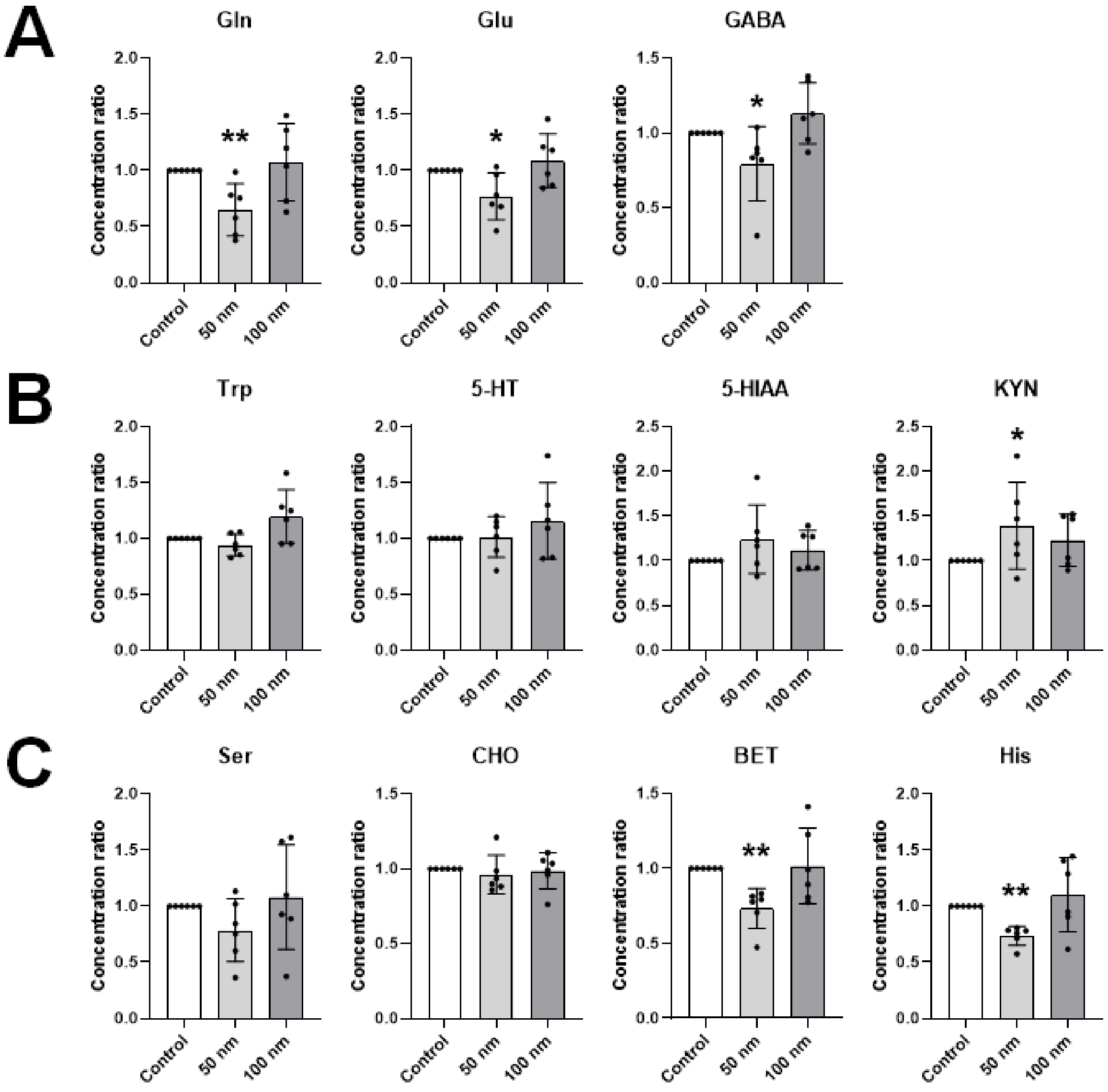

Figure S2. Quantitative analysis of GABAergic (A), serotonergic (B), and cholinergic (C) metabolites in polystyrene nanoparticles (PS-NPs)-exposed zebrafish larvae. Data sets were expressed as the means ± standard error of the mean and statistical significance was set at 0.05, 0.01, and 0.001 (* $p \leq 0.05$, ** $p \leq 0.01$).

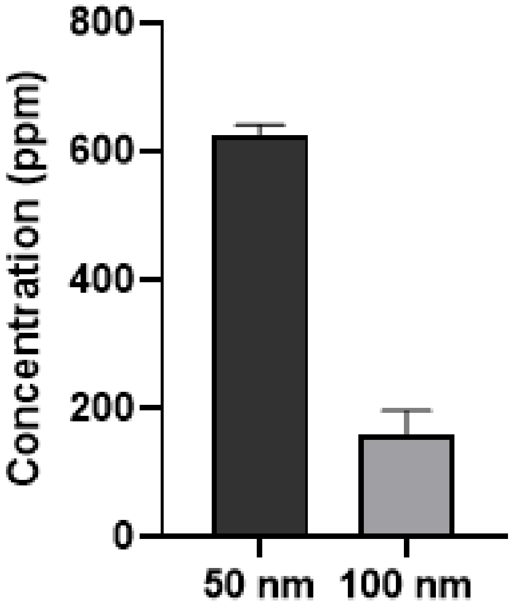

Figure S3. Quantification of accumulated poly-styrene nanoparticles (PS-NPs) in zebrafish larvae.

Table S1. Primers for qRT-PCR of zebrafish genes.

Gene	Primer	Sequence (5′-3′)
actin, beta 1	actb1_FP	CCGTGACATCAAGGAGAAG
	actb1_RP	ATACCGCAAGATTCCATACC
phenylalanine hydroxylase	pah_FP	AGACCAATACTGACTGCCTAC
	pah_RP	AGCTCATGATTTCAACAAGCC
tyrosine hydroxylase	th_FP	GCAGATCGTGTTTGAGGAGAGC
	th_RP	AAACCTTCAAGGTGCGGGACAG
dopa decarboxylase	ddc_FP	ACCACATCTGCTTTCACAC
	ddc_RP	AGCCTGGATTGCTACACAC
catechol-O-methyltransferase a	comta_FP	ACCCTCAGGTGATCTGATCC
	comta_RP	GAGTTTGGTGTCTGGAACG
catechol-O-methyltransferase b	comtb_FP	TCTACAGTTCGCATCGCTC
	comtb_RP	GTCTTCAATTCCAGCCCAG
