# Supplementary figures and images for: Size-Dependent Effects of Polystyrene Nanoparticles (PS-NPs) on Behaviors and Endogenous Neurochemicals in Zebrafish Larvae
Source: Int J Mol Sci. 2022 Sep 14;23(18):10682. doi: 10.3390/ijms231810682 (PMC9505408; doi:10.3390/ijms231810682)

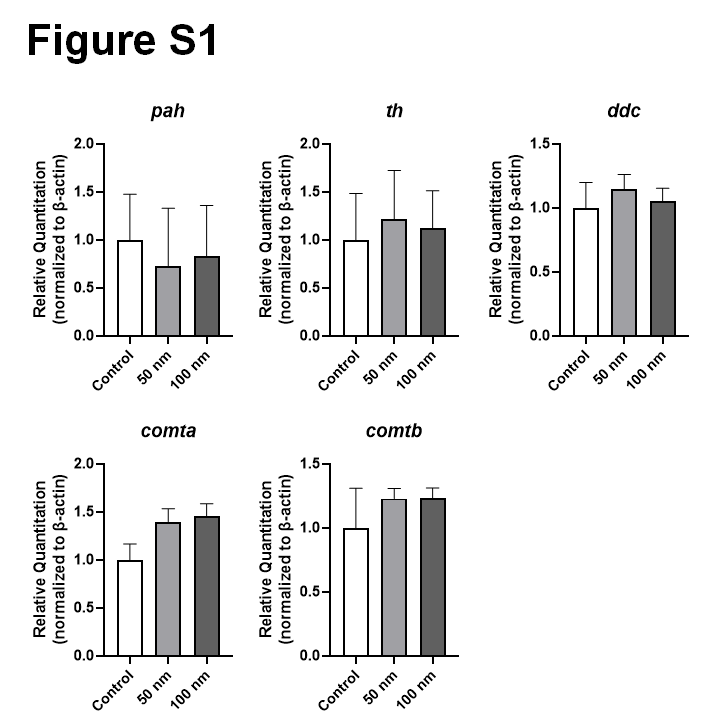

Supplement: Supplementary file 1 [file ijms-23-10682-s001.zip › Figure S1.tif]

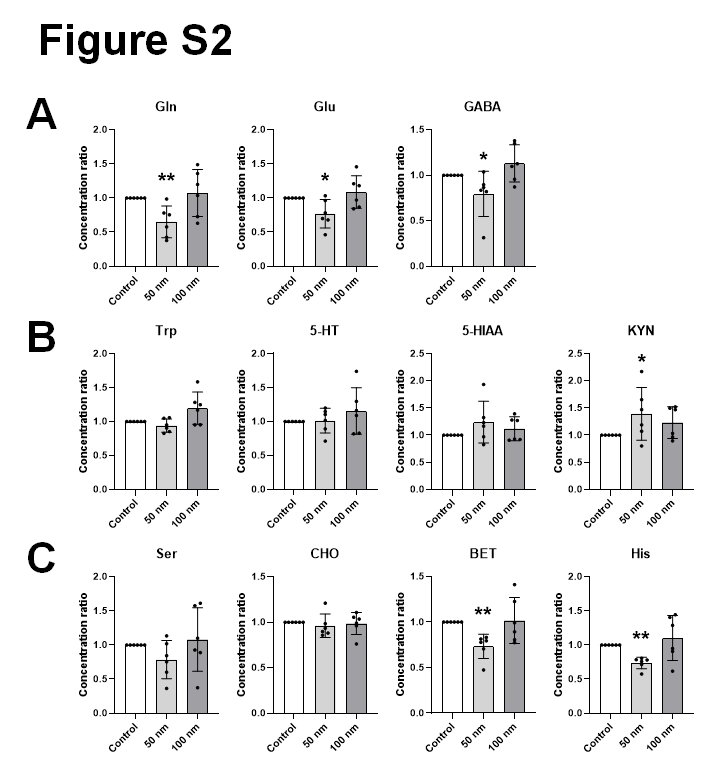

Supplement: Supplementary file 1 [file ijms-23-10682-s001.zip › Figure S2.tif]

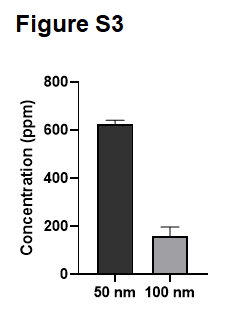

Supplement: Supplementary file 1 [file ijms-23-10682-s001.zip › Figure S3.tif]
